# Assessment of marginal gaps and image quality of crowns made of two different restorative materials: An in vitro study using CBCT images

**DOI:** 10.34172/joddd.2022.039

**Published:** 2022-12-30

**Authors:** Paulo Victor Teixeira Doriguêtto, Daniela de Almeida, Carolina Oliveira de Lima, Ricardo Tadeu Lopes, Karina Lopes Devito

**Affiliations:** ^1^School of Dentistry, Federal University of Juiz de Fora, Juiz de Fora, Minas Gerais, Brazil; ^2^Nuclear Engineering Department, COPPE, Federal University of Rio de Janeiro, Rio de Janeiro, Brazil; ^3^Department of Dental Clinic, School of Dentistry, Federal University of Juiz de Fora, Juiz de Fora, Minas Gerais, Brazil

**Keywords:** CBCT, Dental materials, Gap, Image quality, Zirconia

## Abstract

**Background.:**

The present study assessed the quality of images and the presence of marginal gaps on cone-beam computed tomography (CBCT) images of teeth restored with all-ceramic and metal-ceramic crowns and compared the gap sizes observed on CBCT images with those obtained on micro-CT images.

**Methods.:**

Thirty teeth restored with metal-ceramic and all-ceramic crowns, properly adapted and with gaps of 0.30 and 0.50 mm, were submitted to micro-CT and CBCT scans. Linear measurements corresponding to the marginal gap (MG) and the absolute marginal discrepancy (AMD) were obtained. The objective assessment of the quality of CBCT images was performed using the contrast-to-noise ratio (CNR), and the subjective assessment was defined by the diagnoses made by five examiners regarding the presence or absence of gaps.

**Results.:**

The measurements were always higher for CBCT, with a significant difference regarding AMD. No significant difference in image quality was observed using CNR between the crowns tested. Low accuracy and sensitivity values could be observed for both crowns.

**Conclusion.:**

Marginal mismatch measures were overestimated in CBCT images. No difference in image quality was observed between the crowns. The correct diagnosis of gaps was considered low, irrespective of crown type and gap size.

## Introduction

In cases of extensive loss of tooth structure, indirect restorative techniques are the most indicated for oral rehabilitation. The prosthetic crowns used for this purpose can be made of metal alloys (gold, palladium, silver, cobalt, copper, nickel, or aluminum) or of metal-free materials, such as glass and carbon fibers, resin materials, zirconia-reinforced or all-ceramic materials.^1,2^

The clinical success and longevity of restorations depend, among other factors, on a good marginal fit. The presence of cracks or misfits, also called gaps, is directly associated with fracture, postoperative hypersensitivity, loss of retention due to dissolution of the cementing agent, and the development of secondary caries since they favor microleakage of bacteria and their by-products.^3-5^

Different methods can be used for assessing marginal adaptation.^3^ The marginal fit can be estimated directly by visual and tactile inspection using a probe during a clinical examination.^6^ This can also be assessed indirectly using silicone replicas^7^, optical microscopy, or scanning electron microscopy.^8,9^ Marginal fit can also be assessed radiographically using interproximal, periapical radiographs, cone-beam computed tomography (CBCT),^10-13^ and more recently, by micro-computed tomography (micro-CT).^14-18^

The micro-CT is an in vitro, non-destructive examination that produces high-resolution images and allows different materials to be distinguished in a two- and/or three-dimensional assessment.^4,5^

CBCT is widely used in dentistry for the diagnosis of changes that affect the head and neck region. This exam provides high-resolution 3D images of the scanned regions, in addition to high geometric precision and lower doses of ionizing radiation compared with helical computed tomography.^19^ However, metal dental materials or materials containing high percentages of radiopacifying materials have been related to the formation of artifacts in CBCT images, which decrease the contrast, conceal structures, and, consequently, impair the diagnosis of a given region of interest.^13,20,21^

The ability to distinguish between different structures in the same image is defined as contrast resolution, which, together with the spatial resolution, determines the overall quality of a CBCT image. This property can be objectively assessed by the contrast-to-noise ratio (CNR). The CNR is considered an appropriate physical index to evaluate image quality and has been used in previous studies.^22-25^

 Considering the interference of dental materials with different densities in the final quality of tomographic images and the possible interference of these artifacts in the diagnosis of marginal misfits, the present study assessed the presence of marginal gaps (MGs) in CBCT images of teeth restored with metal-ceramic and all-ceramic crowns and compared the gap sizes observed in CBCT images with those obtained using micro-CT. Furthermore, the quality of CBCT images was objectively assessed by the CNR and subjectively by gaps diagnosed by radiologists.

 The hypotheses tested were: 1) The two tested crowns interfere differently in the quality of CBCT images (CNR) and in the identification of MGs; 2) There are no significant gap size differences in CBCT and micro-CT images.

## Methods

 This was an analytical study with an in vitro experimental design, which was approved by the Research Ethics Committee of the Federal University of Juiz de Fora (UFJF), under protocol number 2.435.835/2017.

###  Sample preparation 

 Thirty sound mandibular molars underwent standardized full-crown preparations using a condensation silicone model (Coltene/Whaledent AG, Altstätten, Switzerland). The teeth were prepared by a single dentist. In the CAD-CAM (computer-aided design - computer-aided manufacturing) digital system (Ceramil Motion, Amann Girrbach, Koblach, Austria). The copings were milled in Zirconia Ceramill ZI White 71L (Amman Girrbach, Koblach, Austria), and the copings in Ceramill Wax Gray 71L (Amann Girrbach, Koblach, Austria), which were subsequently cast in metal. The gaps, measuring 0.3 mm and 0.5 mm, were randomly fabricated on the mesial or distal surfaces, programmed by the CAD-CAM equipment, since the intention was to study the identification of gaps that could be harmful to the integrity of the restoration (subject to infiltration). In the second step, the copings were carefully covered with feldspathic ceramic, taking care not to mask the marginal misfit that was simulated in the previous step.

 The 30 teeth were randomly divided into two groups: 1) All-ceramic group: 15 teeth restored with full crowns, with the zirconia coping being covered by feldspathic ceramic (Kuraray Noritake Dental, Tokyo, Japan) so that ten teeth had marginal misfits (0.3 and 0.5 mm) and five teeth had adequate marginal adaptation; 2) Metal-ceramic group: 15 teeth restored with metal-ceramic crowns composed of the Dan Ceramalloy nickel-chromium alloy (Nihon Shika Kinzoku, Osaka, Japan), covered with the same feldspathic ceramic used in the All-ceramic group. Likewise, ten teeth had a gap (0.3 and 0.5 mm), and five teeth had well-adapted crowns.

Subsequently, the crowns were cemented using the self-etching/self-adhesive resin cement RelyX U200 (3M ESPE, St. Paul, MN, MN) according to the manufacturer’s instructions. After applying the cement to the inner surface of the crowns, the crown/cement set was carefully positioned on each tooth preparation and subjected to a load of 5 N, exerted by a device, for 6 minutes to simulate the definitive clinical cementation procedure and promote the flow and homogenization of the cement film. After removing the rough excess cement, the material was light-cured for 40 seconds on each set surface using an LED light-curing device (Optilight Max, Gnatus, Ribeirão Preto, SP, Brazil) with a light intensity of 1200 mW/cm^2^.

 After cementation, in the teeth restored with full crowns, the mesial surface of the cervical third of the root was identified using a groove equivalent to half the diameter of the active tip of a spherical diamond bur (No.1015) (KG Sorensen, Cotia, SP, Brazil).

###  Acquisition and assessment of micro-CT images

 The restored teeth were scanned using micro-CT (SKYSCAN 1173, Bruker, Kontich, Belgium). To prevent any movement during scanning to capture the images, the samples were stabilized, one at a time, on the equipment fixation device, using utility wax (Technew, Rio de Janeiro, RJ, Brazil), with the mesial surface positioned perpendicularly to the x-ray beam. The following acquisition parameters were used: acceleration voltage: 70 kV; current: 114 µA; 7.90-µm pixel; 1.0-mm aluminum filter; detector matrix: 2240 × 2240 pixels; 0.6º rotation step, and total rotation of 360º, which resulted in an average scanning time of 37 minutes for each sample. These parameters were determined in a pilot study.

 After scanning, the microtomography projections were reconstructed using the NRECON program (Bruker Kartuizersweg 3B, Kontich, Belgium). An ROI (region of interest) with an individualized size encompassing the tooth/restoration interface was established for each tooth. The two-dimensional images, in the TIFF format, corresponding to this ROI, were then reconstructed by the aforementioned software, with the following image parameters: smoothing of 10, ring artifact reduction of 6, and beam hardening correction of 10% for all samples.

 After acquisition and reconstruction, the 3D images in coronal (x-z plane), sagittal (y-z plane), and transaxial (x-y plane) sections were visualized using the DataViewer (SkyScan) software to establish the appropriate mesiodistal position for the later recording of linear measurements. In the sagittal plane, this position was represented when the pre-established groove on the mesial proximal surface could be clearly seen. Once the groove on the mesial proximal surface was located in the DataViewer, all the images of the corresponding sagittal section were saved.

Later, after being saved, the images in the sagittal slices were analyzed in the ImageJ software (U.S. National Institutes of Health, Bethesda, Maryland, USA). When running the slices in this software, the total length of the *gap *(Length_gap_) was determined by subtracting the value in the final slice in which the gap was visualized (Fs_gap_) from the value in the initial slice in which the gap began to form (Is_gap_), where Length_gap_*=*Fs_gap_* – *Is_gap_. Subsequently, the central region of the gap was calculated ([Is_gap_] + [Length_gap_/2]) to provide the midpoint for the measurements. From the midpoint, another four equidistant points, at a distance of every 10 cuts, were chosen, resulting in five points for measurements on each specimen. The equivalence of pixel size in the images was established before starting the measurements.

**Figure 1:**
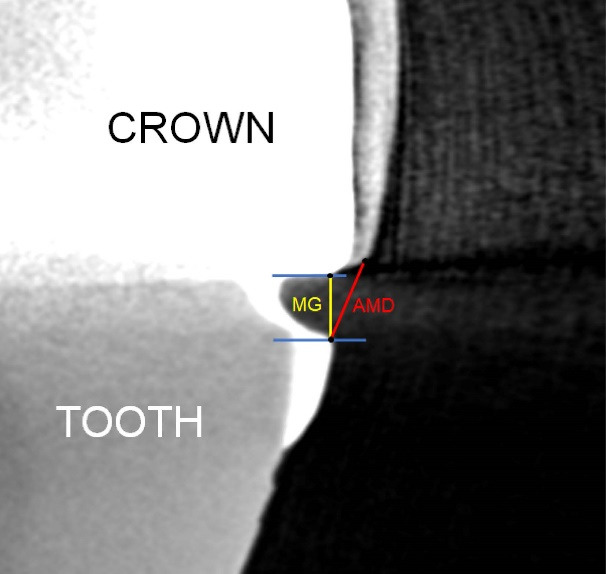
Marginal gap (MG) and absolute marginal discrepancy (AMD) measurements in micro-CT images

The linear measurements corresponding to the MG and the absolute marginal discrepancy (AMD) were defined according to Holmes et al,^26^ where the MG corresponded to the perpendicular distance between the inner surface of the restoration and the bevel at the terminal portion of a prepared tooth. The AMD referred to the distance between the bevel at the terminal part of the prepared tooth and the marginal edge of the restoration, characterizing not only misfit but also marginal overhanging restoration or marginal deficiencies (Figure 1). According to the methodology proposed by Gassino et al,^27^ eighteen random measurements performed in a 360º assessment are necessary to locate misfits in experimental crowns. Since the crown has four surfaces and in this study, the objective was to assess the misfit in one of the proximal surfaces, measurements were made at five equidistant points selected within the total extension of the gap.

 The analyses were performed by the same evaluator using the ImageJ software, at two different time intervals, fifteen days apart, to determine the intra-examiner agreement.

###  Acquisition and assessment of CBCT images

 For the acquisition of CBCT images, an edentulous mandible with alveoli was used. This was fabricated of barium (Nacional Ossos, Jaú, SP, Brazil), which simulated the characteristics of human radiographic bone density. The restored teeth were inserted, one at a time, into the sockets and fixed with utility wax (Technew, Rio de Janeiro, Brazil). Each restored tooth was positioned between two sound teeth, a premolar in the mesial position and a molar located in the distal position, to simulate the point of contact. The buccal and lingual surfaces of the mandible were coated with 15 mm of utility wax (Technew, Rio de Janeiro, RJ, Brazil) to simulate soft tissues.

 Subsequently, the mandible with the teeth duly in position was submitted to CBCT scans using the Orthopantomograph OP300 device (Instrumentarium Kavo Kerr Corp, Tuusula, Finland), with the following technical parameters: field of view (FOV): 5 × 5 cm; 0.085 mm voxel; 90 kV, 6.30 mA, and 8.70 s.

 The CBCT images were analyzed by the same evaluator at two time intervals using the OnDemand3D tomographic image manipulation software (Cybermed, Tustin, CA). Similar to micro-CT images, the central region of the gap was initially calculated, from which another four equidistant slices were established, resulting in five slices for measurements in each specimen. Linear measurements (MG and AMD) were obtained from the proximal surfaces of the restored teeth on the sagittal reconstruction screen, which positioned the reference line along the central long axis of the tooth.

###  CNR measurement in CBCT images

The volumes of each sample were individually loaded into the OnDemand3D Dental software (version 2016.1). In the sagittal and coronal sections, the vertical guideline was positioned longitudinally along the long axis of the restored teeth, and the horizontal guideline was positioned on the tooth‒restoration interface, resulting in the axial reference section, which was captured and exported in BMP format, to be dealt with in the following steps, using the methodology adapted from the study by Rabelo et al.^28^

**Figure 2:**
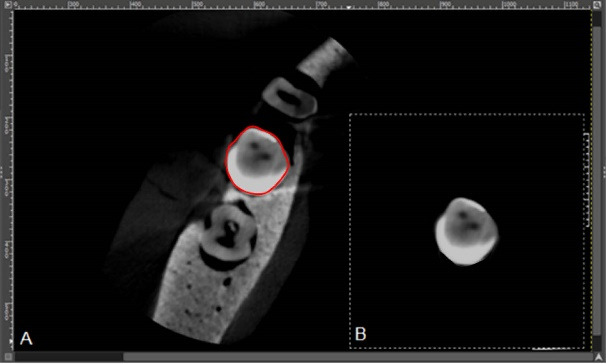
Manipulation of images in the GIMP program. A. Freehand selection of the restored tooth. B. Exclusion of the adjacent images

In the axial image obtained, the region of the tooth was manually delimited using the free selection tool of the GIMP program. The selected structure was cut, transferred to a black background with grayscale (8-bit) in a square format measuring 10 × 10 cm, and exported in JPEG format to proceed with the precise selection of the regions of interest. This cut was necessary, as the pixels of adjacent materials had to be disregarded in the CNR calculation, and it would not have been possible to remove them in the following steps (Figure 2).

**Figure 3:**
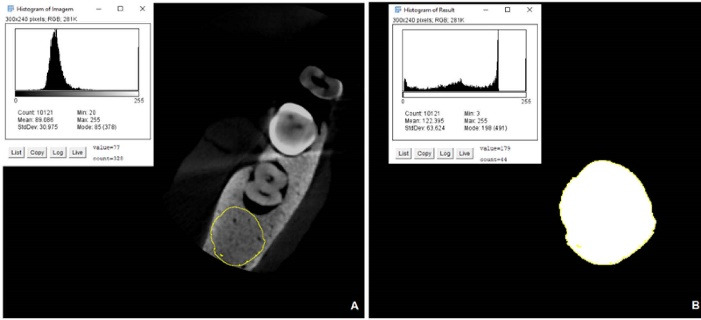
Histogram with data for calculating the CNR. A. Histogram of ROIc. B. Histogram of the restored tooth (ROI_R_)

Subsequently, the images were duplicated and binarized in the Image J software. An ROI with the same size as the region of interest of the restored tooth (ROI_R_) was selected on the outer side of the original acquisition to be used as a control area (ROI_C_). For each ROI, a histogram with the standard deviation (SD) and mean grayscale values was generated (Figure 3).

 Using the values acquired, the CNR of each image could be measured according to the following equation:


$$CNR=\frac{MeanR-MeanC}{\sqrt{\frac{SDR^{2}-SDC^{2}}{2}}}$$

where Mean_R_ corresponded to the average gray values of the ROI of the restored tooth and Mean_C_ to the mean of gray values of the ROI of the control area. SD_R_ and SD_C_ corresponded to the standard deviation of the restored tooth and the control area, respectively.

###  Diagnosis of gaps in CBCT images

 The images of the restored teeth were individually assessed by five oral radiologists experienced in CBCT scans. They were blinded to the materials used to restore the teeth and/or the presence and size of gaps in each tooth.

 The images were assessed using OnDemand3D software (Cybermed, Tustin, CA). The examiners assessed the proximal surfaces (mesial and distal) of each tooth for the presence of gaps in the restored teeth, using a five-score scale: 1: gap definitely present; 2: gap probably present; 3: uncertainty about the absence or presence of a gap; 4: gap probably absent; 5: gap definitely absent.

###  Statistical analysis

The data were analyzed using SPSS (Statistical Package for the Social Sciences, version 21.0, Chicago, USA), with a significance level of 5% (*P* ≤ 0.05). To assess the agreement of quantitative variables, the intraclass correlation coefficient (ICC) was calculated, and the kappa test was used for qualitative variables. The normality of the data was verified by applying the Shapiro-Wilk test. The Mann-Whitney test was used to compare the MG and AMD values between the CBCT and micro-CT examinations and the CNR between the different restorative materials. Sensitivity, specificity, and accuracy values, determined by the area under the ROC curve, were calculated for each crown type and gap size.

## Results

###  Comparison of gap sizes between CBCT and micro-CT images

The ICC indicated an excellent intra-examiner agreement for both MG (0.94; *P* < 0.0001) and AMD (0.95; *P* < 0.0001) values obtained from CBCT images, and for the values obtained in micro-CT (MG: 0.99; *P* < 0.0001; AMD: 0.99; *P <*0.0001).

As the MG (P = 0.038) and AMD (P = 0.024) values did not show normal distribution according to the Shapiro-Wilk test, these variables were compared between the two imaging tests using the Mann-Whitney test. The values were always higher in the CBCT images and showed a significant difference for AMD (Table 1).

**Table 1:** Comparison of MG and AMD values obtained from CBCT and micro-CT images

	**CBCT**		**Micro-CT**		***P*** ** value**
	**Mean (SD)**	**Median**	**Mean (SD)**	**Median**	
MG					
Metal-ceramic	0.37 (0.20)	0.38	0.27 (0.19)	0.21	0.161
All-ceramic	0.39 (0.20)	0.35	0.34 (0.28)	0.25	0.412
Total	0.38 (0.20)	0.36	0.30 (0.23)	0.22	0.114
AMD					
Metal-ceramic	0.66 (0.30)	0.65	0.40 (0.21)	0.39	0.011*
All-ceramic	0.74 (0.39)	0.64	0.41 (0.31)	0.34	0.033*
Total	0.70 (0.35)	0.65	0.41 (0.26)	0.37	0.001*

*Statistically significant difference by the Mann-Whitney test (*P* ≤ 0.05).

###  CNR measurement in CBCT images

As the CNR data did not show a normal distribution according to the Shapiro-Wilk test (P ≤ 0.0001), the Mann-Whitney test was used to compare this variable between the different restorative materials (Table 2). No significant difference was observed between the two crowns.

**Table 2:** Comparison of CNR values obtained from CBCT images of teeth restored with metal-ceramic and all-ceramic crowns

	**Metal-ceramic**		**All-ceramic**		***P*** ** value**
	**Mean (SD)**	**Median**	**Mean (SD)**	**Median**	
CNR	2.36 (1.38)	2.09	2.41 (2.60)	1.45	0.081

###  Diagnosis of gaps in CBCT images

Five evaluators examined the CBCT scans for the presence of gaps; there was significant agreement between them (*P* ≤ 0.05), with kappa values ranging from 0.290 to 0.592.

Sensitivity, specificity, and accuracy values, determined by the area under the ROC curve, were calculated for each crown type and gap size (Table 3). For calculating sensitivity and specificity, the evaluators’ responses were dichotomized into present or absent, with scores 1, 2, and 3 considered the presence of a gap and scores 4 and 5 considered the absence of a gap.

**Table 3:** Mean values of the area under the ROC curve (AUC ROC), sensitivity, and specificity according to each crown type and gap size

		**AUC ROC**	** Sensitivity**	**Specificity**
Metal-ceramic	Gap 0.3 mm	0.21	0.16	0.73
	Gap 0.5 mm	0.28	0.20	0.68
	Total	0.28	0.21	0.68
All-ceramic	Gap 0.3 mm	0.29	0.21	0.53
	Gap 0.5 mm	0.39	0.19	0.48
	Total	0.32	0.21	0.54

 Low accuracy and sensitivity values could be observed for both crowns, irrespective of the gap size. However, the specificity values were higher for metal-ceramic crowns (0.68 to 0.73) than for crowns made of all-ceramic (0.48 to 0.54).

**Figure 4:**
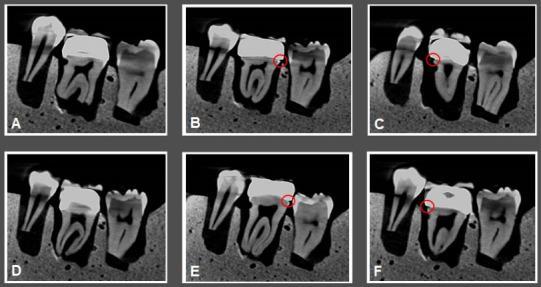
CBCT Images. A. All-ceramic crown without a gap. B. All-ceramic crown with a gap of 0.3 mm. C. All-ceramic crown with a gap of 0.5 mm. D. Metal-ceramic crown without a gap. E. Metal-ceramic crown with a gap of 0.3 mm. F. Metal-ceramic crown with a gap of 0.5 mm

**Figure 5:**
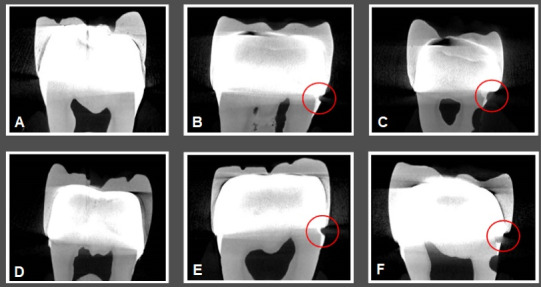
The micro-CT images: A. All-ceramic crown without a gap. B. All-ceramic crown with a gap of 0.3 mm. C. All-ceramic crown with a gap of 0.5 mm. D. Metal-ceramic crown without a gap. E. Metal-ceramic crown with a gap of 0.3 mm. F. Metal-ceramic crown with a gap of 0.5 mm

Figures 4 and 5 illustrate CBCT and micro-CT images obtained from teeth restored with metal-ceramic and all-ceramic crowns.

## Discussion

 The marginal and internal adaptation of dental crowns is closely related to the quality of restorations. Together with the solubility of the luting agent, biofilm accumulation, and conditions of adjacent hard and soft tissues, these factors influence the longevity and success of the prosthetic treatment.

The methods for assessing marginal misfits can be divided into destructive or non-destructive types.^29^ Among the destructive methods, optical microscopy has the highest precision in measurements.^29,30^ Among the non-destructive methods, the following can be mentioned: subjective visual assessment using a millimeter probe; the silicone replica technique, profilometry,^29^ and imaging tests, such as interproximal radiographs, CBCT,^13^ and more recently, micro-CT.^14-18^

CBCT is considered an accurate three-dimensional imaging test for dental diagnostic purposes. Its classic indication covers planning for dental implant surgeries, diagnosis and planning of surgeries to treat impacted teeth and maxillofacial pathologies, detection of bone and dental anomalies, management of dental and maxillofacial trauma, identification of temporomandibular joint disorders, and orthodontic planning.^31,32^ More recently, CBCT has been investigated for its usefulness in the diagnosis of other conditions, including the detection of dental caries and marginal misfits in restorations.^13,33^ Although its initial indication may not be for these purposes, the images that have previously been obtained can be used to assess these dental conditions.

One of the limitations of CBCT, which may justify the low sensitivity and accuracy rates for the diagnosis of MGs, is the formation of artifacts, defined as any image distortion that is not present in the object under analysis.^22,34^ Artifacts are mainly generated by metal or high-density materials by radiation beam hardening that increases the absorption of x-rays.^34^ Consequently, the resulting image is altered by the formation of hypodense bands, hyperdense streaks, and distorted objects, compromising the diagnosis in surrounding areas.^20,35,36^

The hypothesis that there would be no significant differences in gap size between CBCT and micro-CT images was rejected. Overestimation of the MG sizes in the CBCT images, with a significant difference for the AMD, observed in this study, may have been related to the more extensive expression of artifacts in this examination in comparison with the micro-CT, consistent with a study by Mazzi-Chaves et al.^37^ Furthermore, in the CBCT examination, the restored teeth were included in a mandible with two other adjacent teeth; thus, the larger quantity of materials with different densities within the FOV may have resulted in greater beam hardening and consequently, in the more extensive formation of artifacts.^22,38^ Although the image acquisition in micro-CT involved only the individually restored tooth, the beam hardening effect may have been reduced.^37,39^

It is recommended that the quality of images be verified based on four main parameters: spatial resolution, contrast, noise, and the artifacts mentioned above. Spatial resolution or sharpness refers to the ability to discriminate small structures in an image. Contrast is defined by the ability to distinguish fabrics or materials of different densities; however, noise refers to the random variability in voxel values in an image. This variable is subdivided into quantum noise, caused by interactions that occur during the production and attenuation of x-rays and electronic noise, caused by the conversion and transmission of the detector signal. Considered in conjunction, contrast and noise constitute the CNR, an objective physical index useful for assessing image quality in high-density dental structures and materials with variable attenuation levels.^22^

The presence of dental restorative materials of higher densities is known to significantly reduce the CNR of CBCT images. Bayrak et al.^24^ observed that amalgam had lower mean CNR values than compomers. A similar fact was observed in a study by Vasconcelos et al,^23^ in which the CNR showed lower values for the zirconia images, with a significant difference for the titanium images. Gaêta-Araujo et al^40^ observed that teeth with metal intraradicular retainers had lower mean CNR values and a higher number of artifacts, leading to a negative impact on the final image quality. However, in the present study, there was no significant difference in the CNR between the images of the crowns tested, despite the atomic number of zirconium (Z = 40) being relatively higher than the atomic numbers of nickel (Z = 28) and chromium (Z = 24), which were components of the metal alloy used. Thus, the hypothesis that there would be a difference between the two tested crowns was rejected.

Another point contributing to the expression of artifacts was the image reconstruction process. Differently from the software used to manipulate CBCT images, in which the reconstruction process was automatic,^41,42^ the software used for reconstruction of the micro-CT images provided the operator with tools for reducing metal artifacts, with specific filters for correcting beam hardening.^39^

In the present study, the quality of images was assessed not only objectively by CNR but also subjectively, considering the perception of five evaluators concerning the clinical images to diagnose marginal prosthetic misfits. Previous studies have pointed out a strong association between the subjective quality of images and CNR, reinforcing the credibility of this analysis.^43,44^

Considering the examiners’ responses relative to the presence or absence of a gap in the crowns, low accuracy and sensitivity values could be observed for both crowns, irrespective of the size of the gaps. These findings were correlated with the objective analysis, as there was no significant difference in CNR values between metal-ceramic and all-ceramic crowns. The specificity values, however, were higher for metal-ceramic crowns, which could possibly be explained by the lower influence of artifacts in the images of these crowns in comparison with the images of all-ceramic crowns, in which zirconium was the main component.^23,34^

The quality of CBCT images is also known to be changed by some other exposure parameters, such as kilovoltage (kV), milliamperage (mA), FOV, and voxel size.^22,38,45^ It is worth emphasizing that in the present study, a single CT scanner with unique exposure settings was used, which limited the comparison of our results with those obtained with other types of CBCT equipment.

## Conclusion

 It could be concluded that the CBCT images overestimated the marginal misfit measures obtained by the micro-CT, with a significant difference for the AMD. No difference in image quality was observed between metal-ceramic and all-ceramic crowns using the CNR values. The accuracy and sensitivity values were considered low, irrespective of crown type and gap size. CBCT images did not guarantee accuracy in diagnosing MGs in indirect restorations, reinforcing the need for a precise indication of CBCT in Dentistry.

## Funding

 This study was financed in part by the Coordenação de Aperfeiçoamento de Pessoal de Nível Superior – Brasil (CAPES) – Finance Code 001.

## Ethics Approval

 This study was approved by the Research Ethics Committee of the Federal University of Juiz de Fora (UFJF) under protocol number 2.435.835/2017.

## Competing Interests

 The authors do not have any financial interests in the companies whose materials are included in this article. The authors do not have any competing interests.
